# Supplementary material for: Structural basis for IL-1α recognition by a modified DNA aptamer that specifically inhibits IL-1α signaling
Source: Nat Commun. 2017 Oct 9;8:810. doi: 10.1038/s41467-017-00864-2 (PMC5634487; doi:10.1038/s41467-017-00864-2)
Supplement: Supplementary file 1 — Supplementary Information [file 41467_2017_864_MOESM1_ESM.pdf]

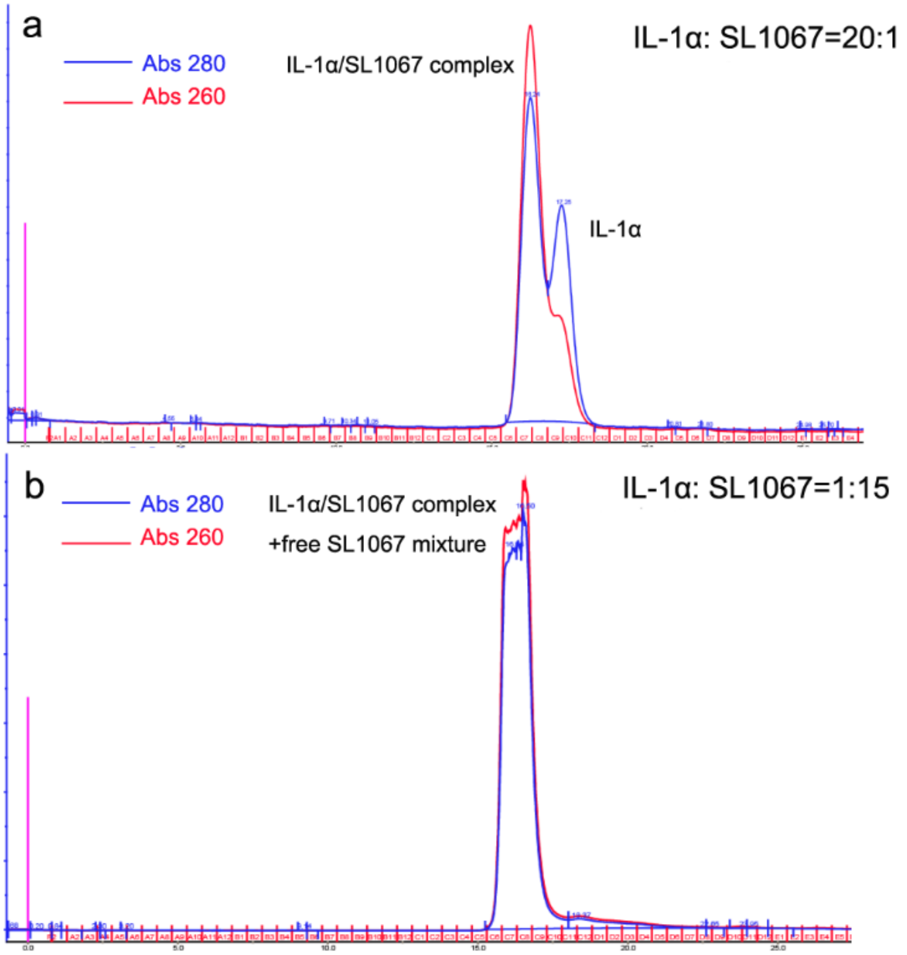

Supplementary Figure 1. Size exclusion chromatography on a Superdex 200 10/300 column determines the ratio of IL-1α and SL1067 in the complex. (a) IL-1α and SL1067 were mixed at a 20:1 ratio and there are two peaks: the left one is the complex and the right one is IL-1α monomer. The elution volume of left peak was calculated to be about one IL-1α plus one SL1067. (b) In excess SL1067, the complex peak is at the same position as shown in (a), suggesting that in solution, IL-1α forms a 1:1 complex with SL1067. Because the UV absorption of SL1067 is much larger than IL-1α, the peak appears to be high and wide, the complex and free SL1067 appear unseparated.

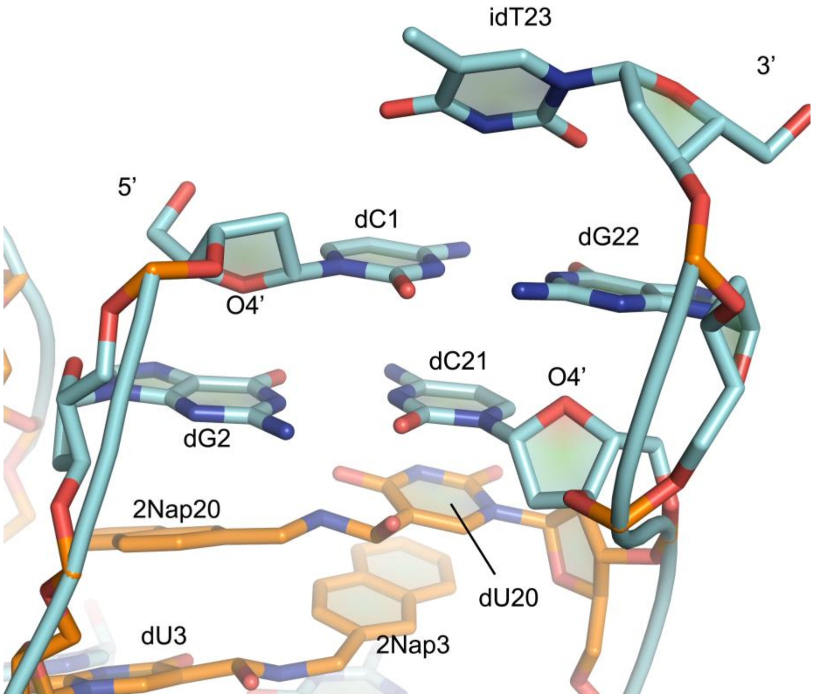

Supplementary Figure 2. Two additional capping interactions: dC1-O4’ and dG2-base, dC21-O4’ and dG22-base.

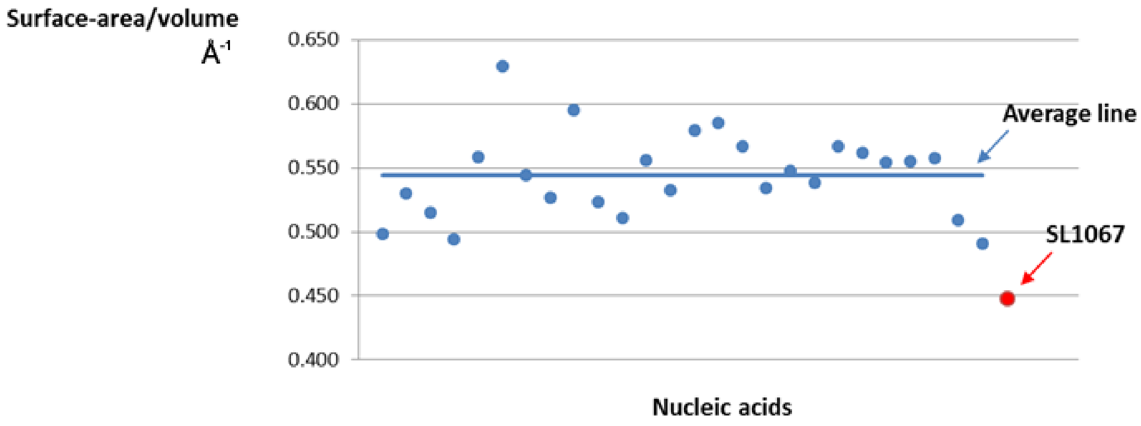

Supplementary Figure 3. Surface-area/volume ratios of the nucleic acids listed in Supplementary Table 3. Previously reported nucleic acids are represented with blue dots and the average value (0.544) is shown with a blue line. The surface-area/volume ratio for SL1067 is shown in red.

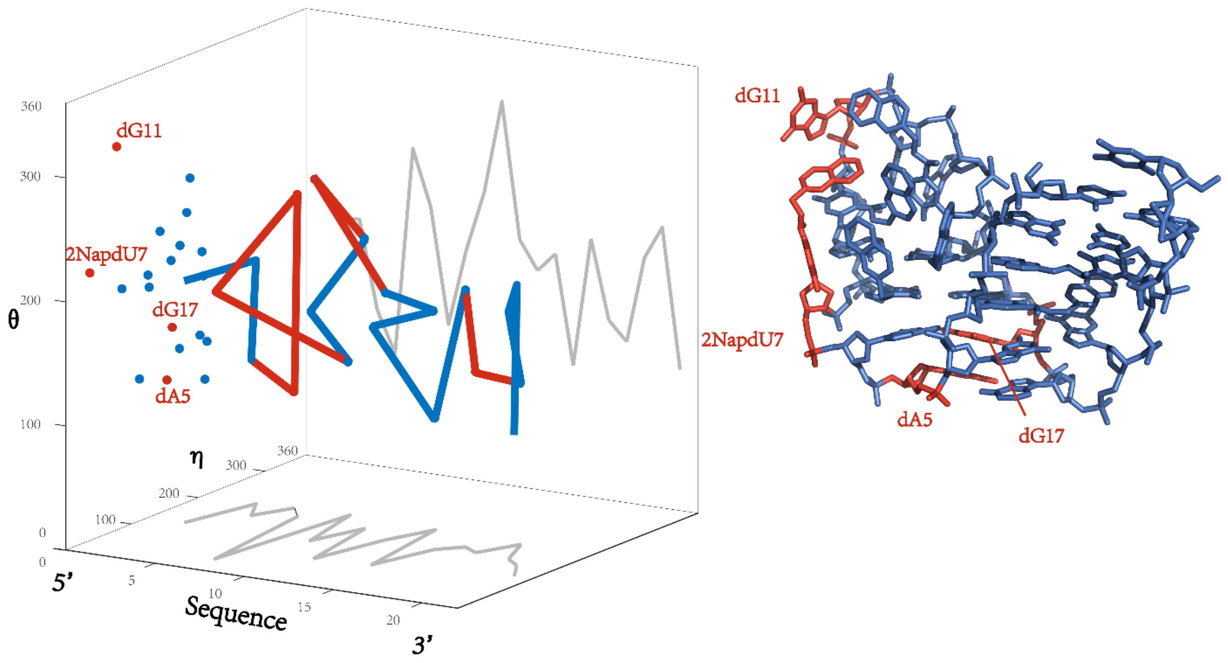

Supplementary Figure 4. A "Worm plot" of SL1067 [1]. The "worm" is plotted by projecting η and θ coordinates along a third sequence dimension. The SL1067 structure (at right) is color-coordinated with corresponding regions of the worm plot. Nucleotides with unusual pseudo-torsion angles are colored red. The plot was generated with Matlab.

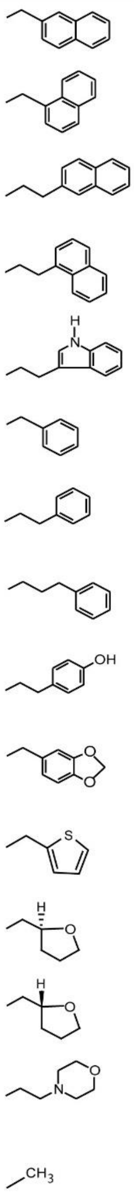

5-dU Modification	3	7	8	10	14	15	20
2Nap-dU	1.0	1.0	1.0	1.0	1.0	1.0	1.0
Nap-dU	1.0	nb	nb	nb	17	nb	1.4
2Ne-dU	nb	nb	nb	nb	nb	nb	27
Ne-dU	12	nb	nb	nb	13	2.5	6.1
Trp-dU	4.7	nb	nb	nb	nb	2.0	16
Bn-dU	1.9	nb	nb	nb	nb	nb	0.49
Pe-dU	nb	nb	nb	nb	nb	20	0.92
Pp-dU	88	nb	nb	nb	12	1.2	1.3
Tyr-dU	nb	nb	nb	nb	nb	nb	6.3
MBn-dU	0.63	nb	1.8	nb	nb	12	1.0
Th-dU	4.4	nb	nb	nb	nb	29	1.1
STHF-dU	nb	nb	nb	nb	nb	nb	nb
RTHF-dU	nb	nb	nb	nb	-	-	-
Moe-dU	nb	nb	nb	nb	nb	nb	22
dT	nb	nb	nb	nb	nb	nb	nb

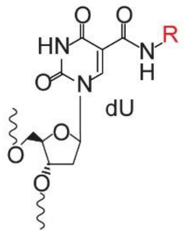

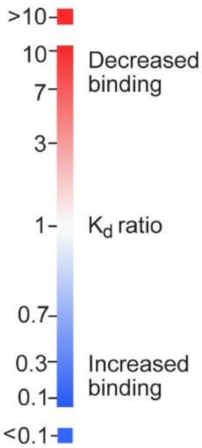

Supplementary Figure 5. Systematic substitution of 2Nap-dU nucleotides in SL1067 with thirteen types of modified dU variants (and with dT). Results of the substitution scanning are expressed as the ratio of K_d values (variant/SL1067). For any variant sequence, K_d ratios > 1 (red) indicate a loss in binding affinity whereas K_d ratios < 1 (blue) indicate an improvement in binding affinity (see color scale bar). (-) indicates no variant was tested; (nb) indicates no binding was detected at an IL-1α concentration up to 100 nM.

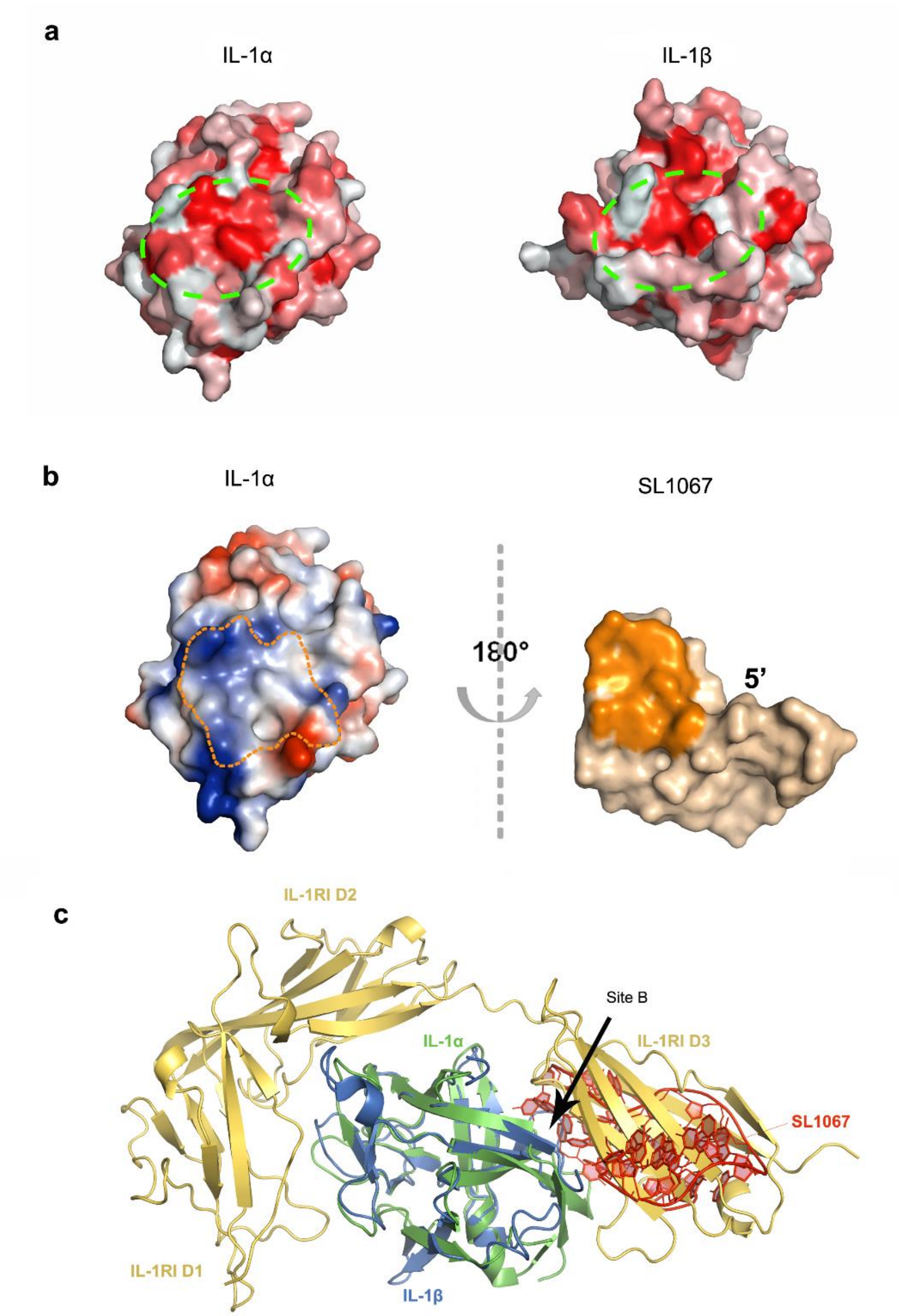

Supplementary Figure 6. (a) Comparison of the hydrophobic surfaces of IL-1α (left) and IL-1β (right), in which the most hydrophobic areas are colored in red (rendered in Pymol). The SOMAmer binding area on IL-1α is circled with a dotted green oval. The corresponding region of IL-1β has a smaller hydrophobic area and a different surface topology. (b) Open-

book view of the IL-1α/SL1067 complex. An electrostatic surface potential rendering of IL-1α shows the small charged areas at the edge of the surface. The IL-1α/SL1067 interface is outlined in orange on IL-1α and the atoms on SL1067 that make contact with IL-1α are colored orange, too. (c) Alignment of the IL-1α/SL1067 complex with the IL-1β/IL-1RI complex (PDB ID 1ITB) shows that the overall structures of IL-1α and IL-1β are similar. The alignment also indicates that SL1067 recognizes the same epitope on IL-1α (site B, black arrow) as Domain III of IL-1RI recognizes on IL-1β. IL-1α is shown in green, SL1067 in red, IL-1β in blue and IL-1RI in yellow.

Supplementary Table 1. Types of base pairs in SL1067.

Base pairs	Type
dC1: dG22	Watson-Crick base pair
dG2: dC21	Watson-Crick base pair
dA9: 2NapdU15	Watson-Crick base pair
2NapdU8: dA16	trans Watson-Crick base pair
2NapdU3: dA16	cis Watson-Crick/Hoogsteen
dG19: dG4	trans Hoogsteen/Watson-Crick
dG4: dG17	trans sugar-edge/sugar-edge
dG17: dG6	trans Watson-Crick/Watson-Crick
dA5: dA18	trans Hoogsteen: Hoogsteen

Supplementary Table 2. Analysis of the surface-area-to-volume ratio for typical DNA RNAs, and protein-binding aptamers. Surface area and volume of each nucleic acid are calculated with Chimera[2].

**Nucleic acid type**	**Protein/aptamer complex**	**PDB ID**	**Surface area Å^2**	**Volume Å^3**	**Surface-area-to-volume ratio Å^{-1}**	**nt**	**Ref.**
Natural RNA	Group II intron Domain 1	4Y1N	33280	66880	0.498	266	[3]
	valyl-tRNA synthetase/tRNA(Val) complex	1GAX	10490	19800	0.530	75	[4]
	Glycyl-tRNA synthetase/tRNA-Gly complex	4KR2	9529	18530	0.514	74	[5]
	5S rRNA	1FFK	16150	32680	0.494	122	[6]
RNA aptamer	MS2 coat protein/RNA aptamer	6MSF	1574	2502	0.629	14	[7]
	NFkB/RNA aptamer	1OOA	4099	7541	0.544	29	[8]
	S8/RNA aptamer	4PDB	5221	9925	0.526	38	[9]
	GRK2/RNA aptamer	3UZS	3134	5273	0.594	19	[10]
	minE Lysozyme/RNA aptamer	4M4O	8154	15580	0.523	59	
	CCL2/L-RNA aptamer	4R8I	5524	10830	0.510	40	[11]
Modified RNA aptamer	thrombin/20-fluoro RNA aptamer	3DD2	3669	6605	0.555	26	[12]
	IgG/20-fluoro RNA aptamer	3AGV	3346	6286	0.532	24	[13]
B-form DNA	reduced AbfR bound to DNA	5HLG	6804	12200	0.558	48	[14]
DNA aptamer	thrombin/DNA aptamer	3QLP	2198	3795	0.579	15	[15]
	vWF/DNA aptamer	3HXO	5925	10140	0.584	40	[16]
	PfLDH/DNA aptamer	3ZH2	3952	6975	0.567	27	[17]
	human alpha-thrombin/DNA aptamer	5CMX	4120	7716	0.534	30	[18]
	HIV-1 reverse transcriptase N-site/DNA aptamer	5I3U	5157	9419	0.548	37	[19]
	human thrombin/two DNA aptamers	5EW1	3664	6815	0.538	26	[20]
			2155	3806	0.566	15	
	human thrombin/two DNA aptamers	5EW2	3686	6562	0.562	25	[20]
			2140	3866	0.554	15	
	HIV-1 reverse transcriptase/DNA aptamer	5D3G	5039	9085	0.555	35	[21]
Modified DNA aptamer	PDGF/modified DNA aptamer (diverse modifications)	4HQU	3978	7141	0.557	24	[22]
	IL-6/modified DNA aptamer (diverse modifications)	4NI7	4799	9437	0.509	32	[23]
	NGF/benzyl modified DNA aptamer	4ZBN	4193	8554	0.490	28	[24]
	IL-1a/Naphthyl modified DNA aptamer	5UC6	3198	7140	0.448	22	

Supplementary Table 3. Statistics* of the sugar pucker, glycosidic torsion and pseudo-torsions of the SL1067 nucleotides. The nucleotides with C3'-endo-like sugar puckers are highlighted in green.

Nucleotide	Pucker type	N-glycosidic bond conformation	Glycosidic torsion angle (χ degree)	η	θ	neighboring p-p distance (Å)
dC1	C2'-endo-like	*anti*	-156.6	-	-	
dG2	C3'-endo-like	*syn*	59.5	123.9	194.8	6.9
2Nap-dU3	C2'-endo-like	*anti*	-92.3	204.9	196.5	6.5
dG4	C2'-endo-like	*syn*	-89.0	171.2	125.5	6.8
dA5	C2'-endo-like	*anti*	-177.3	209.3	92.7	7.0
dG6	C2'-endo-like	*syn*	68.8	187.0	259.6	6.8
2Nap-dU7	C2'-endo-like	*syn*	63.3	36.8	215.1	5.8
2Nap-dU8	C2'-endo-like	*anti*	-153.6	212.4	122.6	6.1
dA9	C3'-endo-like	*syn*	67.9	125.8	184.6	6.8
2Nap-dU10	C2'-endo-like	*syn*	-65.3	182.1	232.9	6.3
dG11	C2'-endo-like	*anti*	-113.2	77.0	308.3	6.7
dG12	C3'-endo-like	*anti*	-129.1	158.6	199.2	6.2
dG13	C2'-endo-like	*anti*	-92.3	206.8	176.5	6.9
2Nap-dU14	C3'-endo-like	*anti*	-154.7	84.9	192.0	5.2
2Nap-dU15	C2'-endo-like	*anti*	-166.4	152.4	104.2	6.1
dA16	C2'-endo-like	*syn*	62.0	171.9	208.5	7.7
dG17	C2'-endo-like	*syn*	-85.1	160.3	145.0	6.3
dA18	C3'-endo-like	*anti*	-162.2	201.9	129.8	6.2
dG19	C2'-endo-like	*anti*	-101.5	156.1	199.5	7.6
2Nap-dU20	C3'-endo-like	*anti*	-113.1	142.0	226.2	6.3
dC21	C2'-endo-like	*anti*	-135.7	111.3	113.7	5.6
dG22	C3'-endo-like	*syn*	69.7	-	-	6.4

* The statistics are calculated with 3DNA[25].

Supplementary Table 4. IL-1α and SL1067 interactions

Hydrogen bonds between IL-1α and SL1067			Hydrophobic interactions between IL-1α and SL1067	
**IL-1α**	**SL1067**	**Length (Å)**	**IL-1α**	**SL1067**
O Ser61	N3 dU7	2.7	Met15	Naphthyl dU10
N Ser61	O4 dU7	2.7		Naphthyl dU15
O Asp65(Na)	O21 2Nap-dU7(Na)	2.4/2.5	Lys60	Naphthyl dU8
NZ Lys60(W4)	O2 dU7(W4)	2.7/2.6	Ile68	Naphthyl dU7
O Trp113	N1 dG11	2.7	Ile18	Naphthyl dU14
N Ile68	O6 dG11	3.2	Trp113	Naphthyl dU14
NE Arg16(H_2O)	O21 2Nap-dU14	2.7/2.4		Base dG11
O Ala66(W3)	O2 dU10(W3)	2.9/2.8		
NE Arg16 (W5)	O21 2Nap-dU14(W5)	2.7/2.4		

Supplementary Table 5. B factor values for atoms surrounding the sodium bonding network, along with the water and the bond lengths to Na.

Atom	B-factor (Å^2)	Bond length to Na (Å) or as indicated
Na	27.79	-
W1	33.16	2.4
W2	43.08	2.6
W3	26.77	2.3
OD1 (Asp64)	31.83	2.4
O (Asp65)	28.74	2.4
O21 (2Nap-dU7)	23.82	2.5
O (Asp64)	28.80	2.8 (to W1)
O2 (2Nap-dU10)	26.43	3.4 (to W2)<br>2.8 (to W3)
O (Ala66)	25.57	2.9 (to W3)
W4	27.74	-
NZ(Lys60)	36.94	2.7 (to W4)
O2(dU7)	26.98	2.6 (to W4)
W5	39.34	-
NE (Arg16)	33.33	2.7 (to W5)
O21 (2Nap-dU14)	31.72	2.4 (to W5)

Supplementary References

1. Duarte CM, Wadley LM, Pyle AM. RNA structure comparison, motif search and discovery using a reduced representation of RNA conformational space. *Nucleic acids research* **31**, 4755-4761 (2003).

2. Pettersen EF*, et al.* UCSF Chimera--a visualization system for exploratory research and analysis. *Journal of computational chemistry* **25**, 1605-1612 (2004).

3. Zhao C, Rajashankar KR, Marcia M, Pyle AM. Crystal structure of group II intron domain 1 reveals a template for RNA assembly. *Nature chemical biology* **11**, 967-972 (2015).

4. Fukai S*, et al.* Structural basis for double-sieve discrimination of L-valine from L-isoleucine and L-threonine by the complex of tRNA(Val) and valyl-tRNA synthetase. *Cell* **103**, 793-803 (2000).

5. Qin X, Hao Z, Tian Q, Zhang Z, Zhou C, Xie W. Cocrystal structures of glycyl-tRNA synthetase in complex with tRNA suggest multiple conformational states in glycylation. *The Journal of biological chemistry* **289**, 20359-20369 (2014).

6. Ban N, Nissen P, Hansen J, Moore PB, Steitz TA. The complete atomic structure of the large ribosomal subunit at 2.4 A resolution. *Science (New York, NY)* **289**, 905-920 (2000).

7. Convery MA*, et al.* Crystal structure of an RNA aptamer-protein complex at 2.8 A resolution. *Nature structural biology* **5**, 133-139 (1998).

8. Huang DB, Vu D, Cassiday LA, Zimmerman JM, Maher LJ, 3rd, Ghosh G. Crystal structure of NF-kappaB (p50)2 complexed to a high-affinity RNA aptamer. *Proceedings of the National Academy of Sciences of the United States of America* **100**, 9268-9273 (2003).

9. Davlieva M, Donarski J, Wang J, Shamoo Y, Nikonowicz EP. Structure analysis of free and bound states of an RNA aptamer against ribosomal protein S8 from Bacillus anthracis. *Nucleic acids research* **42**, 10795-10808 (2014).

10. Tesmer VM, Lennarz S, Mayer G, Tesmer JJ. Molecular mechanism for inhibition of g protein-coupled receptor kinase 2 by a selective RNA aptamer. *Structure (London, England : 1993)* **20**, 1300-1309 (2012).

11. Oberthur D*, et al.* Crystal structure of a mirror-image L-RNA aptamer (Spiegelmer) in complex with the natural L-protein target CCL2. *Nature communications* **6**, 6923 (2015).

12. Long SB, Long MB, White RR, Sullenger BA. Crystal structure of an RNA aptamer bound to thrombin. *RNA (New York, NY)* **14**, 2504-2512 (2008).

13. Nomura Y*, et al.* Conformational plasticity of RNA for target recognition as revealed by the 2.15 A crystal structure of a human IgG-aptamer complex. *Nucleic acids research* **38**, 7822-7829 (2010).

14. Liu G*, et al.* Structural Insights into the Redox-Sensing Mechanism of MarR-Type Regulator AbfR. *Journal of the American Chemical Society* **139**, 1598-1608 (2017).

15. Russo Krauss I, Merlino A, Giancola C, Randazzo A, Mazzarella L, Sica F. Thrombin-aptamer recognition: a revealed ambiguity. *Nucleic acids research* **39**, 7858-7867 (2011).

16. Huang RH, Fremont DH, Diener JL, Schaub RG, Sadler JE. A structural explanation for the antithrombotic activity of ARC1172, a DNA aptamer that binds von Willebrand factor domain A1. *Structure (London, England : 1993)* **17**, 1476-1484 (2009).

17. Cheung YW, Kwok J, Law AW, Watt RM, Kotaka M, Tanner JA. Structural basis for discriminatory recognition of Plasmodium lactate dehydrogenase by a DNA aptamer. *Proceedings of the National Academy of Sciences of the United States of America* **110**, 15967-15972 (2013).

18. Chen Z, Yang H, Pavletich NP. Mechanism of homologous recombination from the RecA-ssDNA/dsDNA structures. *Nature* **453**, 489-484 (2008).

19. Das K, Balzarini J, Miller MT, Maguire AR, DeStefano JJ, Arnold E. Conformational States of HIV-1 Reverse Transcriptase for Nucleotide Incorporation vs Pyrophosphorolysis-Binding of Foscarnet. *ACS chemical biology* **11**, 2158-2164 (2016).

20. Pica A*, et al.* Through-bond effects in the ternary complexes of thrombin sandwiched by two DNA aptamers. *Nucleic acids research* **45**, 461-469 (2017).

21. Miller MT, Tuske S, Das K, DeStefano JJ, Arnold E. Structure of HIV-1 reverse transcriptase bound to a novel 38-mer hairpin template-primer DNA aptamer. *Protein science : a publication of the Protein Society* **25**, 46-55 (2016).

22. Davies DR*, et al.* Unique motifs and hydrophobic interactions shape the binding of modified DNA ligands to protein targets. *Proceedings of the National Academy of Sciences of the United States of America* **109**, 19971-19976 (2012).

23. Gelinas AD*, et al.* Crystal structure of interleukin-6 in complex with a modified nucleic acid ligand. *The Journal of biological chemistry* **289**, 8720-8734 (2014).

24. Jarvis TC*, et al.* Non-helical DNA Triplex Forms a Unique Aptamer Scaffold for High Affinity Recognition of Nerve Growth Factor. *Structure (London, England : 1993)* **23**, 1293-1304 (2015).

25. Lu XJ, Olson WK. 3DNA: a versatile, integrated software system for the analysis, rebuilding and visualization of three-dimensional nucleic-acid structures. *Nature protocols* **3**, 1213-1227 (2008).
